# Telehealth in Informal Caregivers of Stroke Survivors: A Systematic Review

**DOI:** 10.3390/jcm13061810

**Published:** 2024-03-21

**Authors:** Juan Carlos Zuil-Escobar, Jose Antonio Martín-Urrialde, Juan Andrés Mesa-Jiménez, Rocío Palomo-Carrión, Carmen Belén Martínez-Cepa

**Affiliations:** 1 Departamento de Fisioterapia, Facultad de Medicina, Universidad San Pablo-CEU, CEU Universities, Urbanización Montepríncipe, 28668 Alcorcón, Spain; jamurria@ceu.es (J.A.M.-U.); jmesaj@ceu.es (J.A.M.-J.); 2 Physiotherapy Department, Universidad de Castilla-La Mancha, 45071 Toledo, Spain; rocio.palomo@uclm.es

**Keywords:** caregiver, caregiver burden, stroke, telemedicine

## Abstract

**Background**: There has been an increase in people with disabilities who require continuous care, which often falls to informal carers (ICs). Stroke is one of the conditions where ICs are most needed. Therefore, it is necessary for ICs to improve their caregiving skills and self-care capacity. Telehealth (TH) can facilitate them. The aim of this systematic review is to summarize the evidence of the effects of interventions on ICs of stroke patients. **Methods**: The search was conducted in Pubmed, Scopus, Web of Science, CINALH, Psychology and Behavioral Sciences Collection, and APA PsycInfo. Key search terms included “stroke”, “informal caregiver” and “telemedicine”. Only randomised clinical trials were included. **Results**: A total of 2031 articles were found in the databases, 476 were screened and 19 clinical trials met the eligibility criteria. Different TH programmes have evaluated many outcomes related to physical and emotional health. The TH tools included phone, videophone, web-based interventions, and social media. The most investigated outcome was depression; although contradictory results were found, the TH may have helped to prevent an increase in depressive symptoms. There were inconsistent results on the caregiving burden and the preparedness of the IC. However, TH has positive effects on the health of the ICs, reducing the number of unhealthy days, anxiety, task difficulty perception, and improving psychological health. **Conclusions**: TH may be a useful tool to improve the abilities and health of ICs of SS. No adverse effects have been reported. More quality studies evaluating the effects of telemedicine on the ICs of stroke survivors, as well as the most appropriate doses, are needed.

## 1. Introduction

In recent years, there has been an increase in life expectancy, which will continue in the coming decades [1]. Globally, the number of people aged 60 and over will increase by 56% by 2030, and by 2050, the global elderly population is expected to reach 1.5 billion [2]. Longer life expectancy has led to an increase in the number of chronically ill and disabled people [3]. Their management has become a challenge for society and the healthcare system.

Dependent persons require continuous care over time, which is often provided by an informal caregiver (IC) [4]. An IC is a person responsible for the unpaid care of a sick or elderly person who is unable to perform the activities of daily living on their own [5]. They provide physical, emotional, and sometimes financial support, and spend most of the day with them [6,7]. The IC is usually a close family member [7] who has no specialised training [8]. However, caregiving takes a significant toll on ICs, affecting both their physical and psychological health, as well as their social, professional, and economic spheres [9,10,11]. The IC often puts the needs of the person they care for before their own personal needs and lifestyle preferences [12]. Half of them report some form of caregiving overload [13] and 17% suffer from significant health problems, particularly neuropsychiatric disorders [14], which also affect their ability to care [4].

In the United States, it is estimated that there are 17.7 million people providing informal care to people over the age of 65 years [7]. There is therefore a need to develop interventions to support them in their role and improve their health. Although ICs are aware of the need to look after their health, many find it difficult to find the time to do so or to follow the advice given by healthcare professionals [15].

Stroke is the third leading cause of disability worldwide [16] and its prevalence continues to increase [17]. One-third of stroke survivors (SS) suffer permanent disability [18], while 40% of SS require care, with the IC playing a key role in their recovery [19] and in preventing further episodes [20]. The ICs of SS do not have the skills to manage the disease [21]. In addition, most of these ICs are older people with other health problems [22]. For example, up to 50% of them show depressive symptoms, a percentage that rises to 80% in the acute phase of the disease [23]. The perceived burden of IC is mainly determined by the functional status of the caregiver, the duration of care, the number of hours of caregiving per day, and self-efficacy [24]. One of the challenges for health systems and society is to improve the knowledge of ICs about the pathology and their ability to care, as well as to provide them with adequate tools for self-care [25]. It is therefore necessary that ICs in stroke have resources available to them during the post-discharge process, as well as regular communication and support from healthcare professionals [26].

Telehealth (TH) can help in the workplace [27] by facilitating access to services without the need to travel, improving adherence to interventions and facilitating care [28]. For ICs, it helps them by reducing absenteeism [12] and financial costs and facilitating access to interventions [29]. The TH has been shown to have a positive effect on the emotional and psychosocial state of ICs in chronic patients [4,12]. In the case of Alzheimer’s disease, TH has been shown positive effects on the health of ICs, helping them with the disease process and in their caregiving role [30].

Several TH programmes have been used for stroke. Therefore, telerehabilitation programmes have shown similar results to conventional rehabilitation programmes in improving patients’ activities of daily living, motor function, balance, and quality of life [31,32]. In addition, two systematic reviews on the use of telerehabilitation in stroke care showed that these programmes have positive health outcomes for ICs [33,34].

TH can help solve some of the problems faced by ICs of stroke patients. They use internet tools, such as Google, to search for information about their unmet needs and to connect with other caregivers [35]. Similarly, there are also a number of mobile applications available to support these ICs. A scoping review found that these apps provide help in three main areas: caregiver support, barriers, and informal caregiver support [36]. In addition, the mHealth tools for ICs of stroke survivors include several functionalities such as information resources, risk assessment, remote monitoring, data sharing or reminders [37]. However, no systematic reviews were found that evaluated the effects of TH programmes specifically targeted at ICs of stroke survivors.

The aim of this systematic review is to summarize the evidence of the effects of interventions on ICs of patients who have suffered a stroke.

## 2. Materials and Methods

### 2.1. Information Sources

A search was conducted to identify relevant papers evaluating the effects of TH on ICs of SS in the following databases: Web of Science, Scopus, Pubmed, CINAHL, Psychology and Behavioral Sciences Collection, and APA PsycInfo. The systematic review was conducted according to the PRISMA principles [38]. This systematic review was not registered in any public database.

### 2.2. Eligibility Criteria

The search strategy includes terms related to IC, TH, and stroke. The following inclusion was defined by criteria according to the PICO framework [39]:

Population: IC of SS. Both males and females were included.

Interventions: TH interventions in IC of SS.

Comparison: conventional interventions.

Outcomes: indicators of caregiver burden (fatigue, burnout, etc.), physical and emotional health (depression, general health, quality of life), self-efficacy, etc.

In terms of study design, only randomised clinical trials were included. Conference abstracts were excluded, as were reviews and books. There were no time restrictions applied, and articles published in both English and Spanish were included. If the interventions focused on SS, although the ICs were involved, the studies were also excluded.

### 2.3. Search Strategy

Concerning the search keywords, terms (both MeSH and plain language) related to “stroke”, “informal caregiver” and “telehealth” were used.

The following filters were used in the different databases:

- Pubmed, article type: “clinical trial”, “controlled clinical trial”, “randomized clinical trial”; language: “English”, “Spanish”.
- Web of Science: “English”, “Spanish”, “clinical trial”.
- Scopus: “English”, “Spanish”, “article”.
- CINALH, Psychology and Behavioral Sciences Collection and APA PsycInfo: “English”.

Appendix A shows the search strategy for each database.

### 2.4. Selection Process and Data Collection

After performing the bibliographic search in the above-mentioned databases, we used the filtering tools of each of them to eliminate the articles that did not meet the inclusion criteria. In addition, we searched the selected articles for relevant bibliographical references. We also searched for the results of the identified clinical trial protocols.

All references were exported to Mendeley Reference Manager, where duplicates were removed. Next, the titles and abstracts of the included articles were read and those that did not meet the objectives or criteria of the study were excluded. Finally, the full text of the selected articles was read and those that did not meet the objectives of the systematic review or did not meet the inclusion criteria were excluded.

A PRISMA flowchart [38] was used to organize the information, including the number of studies removed by the automatic tools of the databases, duplicates, and selected studies. The initial selection of articles was carried out by two independent reviewers according to the defined inclusion criteria, based on the reading of titles and abstracts only. Subsequently, the articles that were selected or for which the title and abstract did not provide sufficient information underwent a second phase of full-text review. The two reviewers performed the assessment independently according to the eligibility criteria. A consensus meeting was held in case of disagreement.

### 2.5. Data Extraction

Data extraction was performed using an Excel form designed for this systematic review. One independent reviewer extracted data, including article information, population information, interventions, comparisons, outcomes, results, and conclusions. A second reviewer checked all the extracted data. A consensus meeting was held if there was disagreement between the reviewers.

A critical review of the included articles was performed, analysing the main aspects: participants (age, gender), interventions, comparisons, outcome measures, and results. In terms of outcomes, variables related to caregiver burden (stress, burnout, burden, strain, etc.), physical (general health, unhealthy days, etc.), mental health (depression, anxiety, etc.), quality of life, ability to cope with caregiving tasks and self-perception of caregiving were collected.

### 2.6. Risk of Bias

The risk of bias was assessed using version 2 of the Cochrane risk-of-bias tool for randomised trials (RoB 2) [40]. This tool measures the risk of bias in the results of randomised trials. To identify potential bias in the results, this tool is divided into five domains:

(1) Bias arising from the randomisation process.
(2) Bias due to deviations from the intended interventions.
(3) Bias due to missing outcome data.
(4) Bias in the outcome measurement of the outcome.
(5) Bias in the selection of the reported outcome.

A separate score is given for each domain, and an overall score is calculated. The final rating is “low risk” when all domains indicate low risk. If there are concerns in any domain, the final rating will reflect “some concerns”. If there is a high risk in any domain, the overall result is categorised as “high risk”.

Two independent reviewers assessed the methodological quality and the risk of bias in the selected articles.

### 2.7. Quality Assessment

The JADAD scale was used to assess their methodological quality. This scale consists of five items and RCTs are of good quality if they score 3 or higher [41]. This scale has been shown to have good reliability [42].

### 2.8. Data Synthesis

A narrative synthesis of the data was carried out. Descriptive tables were used to compare the studies, including sample size, age and gender of participants, characteristics of interventions, duration of interventions, outcome measures, follow-up, and main results. In the case of missing data, the authors were contacted to obtain unreported data. Effect measures included Student’s *t*-test and ANOVA or non-parametric equivalent tests.

The review was carried out from December 2023 to February 2024.

## 3. Results

### 3.1. Study Selection

**Figure 1:**
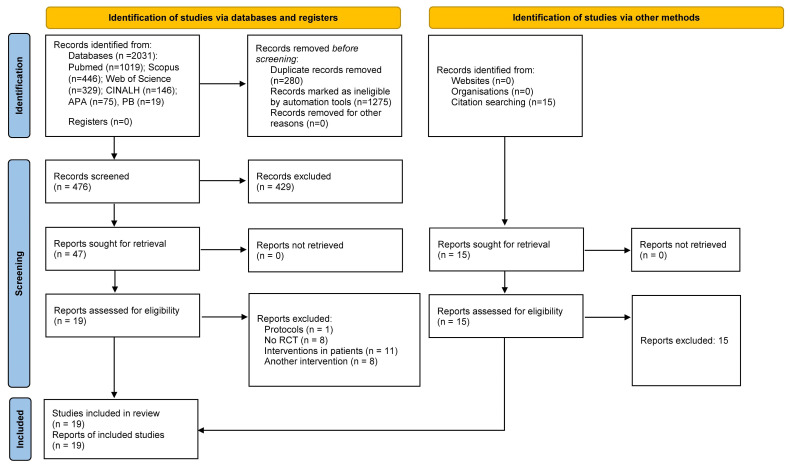
Prisma Flowchart showing the review process.

Figure 1 shows the PRISMA flowchart describing the selection process for this systematic review. Two thousand and thirty-one preliminary articles were found in the databases. After removing duplicates and records marked as ineligible by automated tools, 476 articles were scanned for title and abstract, and 429 were excluded. A total of 47 articles were read in full, and 28 articles were excluded. Nineteen articles [43,44,45,46,47,48,49,50,51,52,53,54,55,56,57,58,59,60,61] met the eligibility criteria and were included in the final review. No further articles were found by searching other sources.

### 3.2. Sample Size and Participant Characteristics (Age and Number of Women per Study) of the Articles Reviewed

A total of 1483 ICs were included in all the articles reviewed. Table 1 shows the main characteristics of the population included in the articles reviewed.

The largest sample size was for the article by Goudarzian et al. [54], which included 152 ICs. On the other hand, Grant et al. [43] included 30 participants.

In terms of gender, the majority of the ICs are women [43,44,45,46,47,50,51,52,53,54,55,56,58,59,60,61] and in most articles, the IC was the spouse or the daughter/son of the SS [43,44,45,47,49,50,52,55,60]. The average age of the ICs is over 50 years, with the exception of Goudarzian et al. [54] and Mohammadi et al. [61], Mou et al. [57], and Hussin et al. [60], where it is over 45 years. Only Elsheikh et al. [56] and Demir et al. [59] included ICs below 40 years old.

### 3.3. Interventions

Table 2 shows the characteristics of the interventions and the outcomes of the articles included in this systematic review. In terms of interventions, the reviewed articles included specific intervention programmes using telephone [43,44,45,50,51,52,53,54,55,57,58,59,61], videophone [47], web-based interventions [46,48,49,52,59], mobile applications [60], and social media [61].

Comparisons included home visits [43], sham telephone calls [44,52], usual care [44,45,46,47,49,51,53,54,55,57,58,59,60,61], online basic information [48], and information letters [50] and booklet [56].

In addition to the heterogeneity of the interventions, there was also wide variation in the duration of the interventions and the follow-up.

### 3.4. Outcomes

The interventions focused on different aspects of the CI, and the studies that were reviewed were concerned with the effects of interventions on the health of ICs. Depression was one of the most important outcomes evaluated [43,44,45,46,47,48,50,51,52,53,55]. General health perceptions were assessed in six articles [43,44,45,53,59,61]. The burden of the care was also evaluated in nine articles [43,44,47,53,55,56,57,58,59]. Changes in the lifestyle of the CIs have also been studied [60].

Other outcomes assessed were the CI’s ability to cope with the tasks [43,44,45,53] and the caregiver’s preparedness [43,44,48,49,50,53,58,59], satisfaction [43,44,46,47], and functional independence [51]. The impact of caregiving on the life of the CI [45,56], the number of unhealthy days [52], self-esteem [48], threat appraisal [45], anxiety [53,54] or the caregiver’s stroke risk [60], social support [48,53], and the family functioning were also evaluated [51,57].

### 3.5. Results

Table 3 shows the main outcomes of the studies.

Depression was assessed in most of the articles. Some articles showed that the TH interventions led to an improvement in the depressive symptoms of the CIs compared to control interventions [43,44,48,50,52,55]. However, other articles found no significant improvement [45,46,47,51,53,54].

There are conflicting results regarding caregiver burden. Perrin et al. [47] showed that the intervention reduced burden at 3 months and LeLaurin et al. [55], Mou et al. [57], and Betik et al. [58] also found a reduction in burden. However, Grant et al. [43,44] and Elsheikh [56] did not find a reduction in caregiver burden.

The TH has a positive effect on the lives of CIs, reducing the number of unhealthy days [52], anxiety [54], functional independence and family functioning [51], and increasing lifestyle changes [60]. In terms of general health perception, only Mohammady et al. [61] found an improvement in the psychological domain of the SF-36.

### 3.6. Risk of Bias

**Figure 2:**
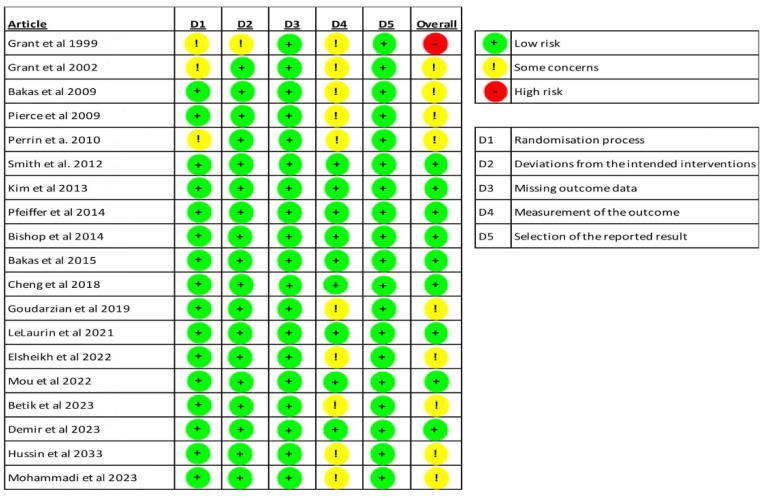
RoB assessments for each study. The plus sign means low risk or bias; the question mark moderate RoB and minus sign denotes high risk of bias and [43,44,45,46,47,48,49,50,51,52,53,54,55,56,57,58,59,60,61].

Nine articles have a low risk of bias [48,49,50,51,52,53,55,57,59], nine have some concerns [44,45,46,47,54,56,58,60,61] and one study has a high risk of bias [43]. Figure 2 shows the results of the RoB2 scale.

### 3.7. Quality Assessment

Sixteen of the articles included in this systematic review are of good quality [45,46,47,48,49,50,51,52,53,55,61], with a score of 3 on the JADAD scale. Only three articles were of low methodological quality, two with a score of 2 [44,54] and one with a score of 0 [43]. Appendix B shows the JADAD Scale.

## 4. Discussion

Nineteen clinical trials were included in this systematic review. It should be noted that a variety of TH tools were used, in particular the use of telephone calls; video conferencing, messaging, and mobile applications were also used. These interventions may be helpful to ICs to support them in their caregiving role. In all cases, these systems have shown no worse results than conventional interventions, and in some trials have been superior to them. No adverse effects have been reported.

ICs need tools and interventions that enable them both to improve their capacity to provide care and to improve their own health [62]. Therefore, it is necessary that ICs of SS have resources available to them during the post-discharge process, as well as regular communication and support from health professionals [26].

The use of TH in IC can serve several purposes: education, counselling, therapy, social support, and data monitoring [4,27]. There are several TH technologies that can be used to perform these functions, including telephone calls, videoconferencing, text messaging, and the use of web-based interventions or mobile applications [4]. There is also a wide choice of providers. Piran et al. [63] identified 843 mobile applications on iTunes in 2019. These apps had different functions: communication, stroke risk calculation, speech therapy, motor recovery, etc. Lobo et al. [37] identified 47 apps for IC stroke survivors. They focused on several functionalities, such as educational resources, risk assessment, remote monitoring, data sharing, and reminders. However, there was no single application that covered all these aspects.

### 4.1. Population

One of the most important issues in the design and use of HT in SS ICs is the consideration of their demographic characteristics. Previously, it has been noted that in most cases, the IC is a close relative, with this role mainly falling to the spouse and/or children of the caregiver [7]. In the studies included in this systematic review, the CI has a direct family relationship with the SS, being mainly the spouse or daughter/son [43,44,45,47,49,50,52,55]. In terms of age, previous studies have shown that the ICs are usually older people [22], as we found in our systematic review; ICs were over 45 years old, with the majority of articles being over 50 years old [43,44,45,46,47,48,49,50,51,52,55,58]. And it is worth noting that there are more women than men in most studies [43,44,45,46,47,50,52,53,54,55,58,59,60,61].

ICs do not have specialised training to perform their role [7], but they are willing to improve their caring function [8]. This may also be one of the points where TH can help the ICs.

### 4.2. Interventions

The studies reviewed included specific care programmes for ICs using different types of TH, including telephone [43,44,45,50,51,53,56,57,58,59], videophone [47], web-based interventions [46,48,59], mobile applications [60], and social media [61]. ICs’ familiarity with TH tools may facilitate their acceptance, thereby improving adherence to these interventions. However, even if ICs are unfamiliar with the technology, they can adhere to the programme properly if they receive the right support [46]. TH can improve the interventions received by ICs by facilitating access, limiting travel problems, and improving adherence to programmes [28].

In addition to TH, the studies reviewed included specific care programmes for ICs, aimed at meeting their needs. Thus, the use of the TH tool in isolation, without including a programme tailored to the needs of the ICs, did not show significant effects on some of the CGs [45,48,52]. When the programme allows the IC to contact a health professional [54], adherence is higher than 96%.

Although new technologies can be challenging for ICs [64], many ICs find that TH helps them in their care work [65], offering new opportunities for care and improving their own health [66]. It is also noteworthy that the ICs find TH to be very positive and useful and recommend its use [67,68,69,70]. In fact, in some cases, they have higher ratings than face-to-face interventions [71]. Even if users have no previous experience, they can use the tools if there is adequate technical support [69,70,72,73]. Users should be given appropriate information, and it should not be assumed that they are familiar with the tools, even if they are similar to those they use in their daily lives [73].

### 4.3. Outcomes

The articles included in this systematic review assessed several outcomes. One of the most important outcomes was depression. Depressive symptoms were assessed in most articles, using the Centre for Epidemiologic Studies Depression Scale [43,44,46,47,48,50,53,55]. Other instruments used were the Patient Health Questionnaire Depression Scale [45,48,52] or the Beck Depression Index [54]. Caregiving burden was assessed using the Caregiving Burden Scale [43,44], the Caregiver Strain Index [47,53], the Zarit Burden Interview-Short Form [55,56,58] or the Caregiver Burden Inventory [57].

Self-perceived general health was assessed using one instrument, the SF-36 [43,44,45,61] or the SF-12 [53]. The WHO-Quality of Life was used in one article [56]. However, for other outcomes, the articles used different instruments. For example, the ability of the CI to cope with the tasks was assessed using the problem-solving inventory [43,44,53] or the Perceived Difficulty with Tasks [45]. Caregiver preparedness was assessed using the Preparedness for Caregiving Scale [43,44,59], the Mastery Scale [48], the Caregiving Mastery Scale [49], the Caregiving Competence Scale [53,59], and the Sense of Competence Questionnaire [50]. IC satisfaction was assessed using the Client Satisfaction Questionnaire [43,44], the VA Care Coordination and Home Telehealth Patient Satisfaction Survey [47] and the Satisfaction with Life Scale [46]. The fact that the reviewed articles used different assessment tools makes it difficult to compare their results. The anxiety was evaluated using the Beck Anxiety Inventory [54] or the generalised anxiety disorder scale-7 [57]. Social support was also evaluated [48,53] as well as family functioning [51,53].

Some outcomes were only assessed in one article: the impact of caregiving on the life of the CI (using the Caregiving Outcomes Scale) [45], the number of unhealthy days [52], self-esteem [48], threat appraisal [45], functional independence [51] or the caregiver´s stroke risk [60].

### 4.4. Results

One of the most investigated outcomes in the articles was depression. The results found were contradictory, with some studies showing a reduction in depressive symptoms [43,44,48], and others not [45,47]. Of particular interest are the results of Pfeiffer et al. [50] and Bakas et al. [52], who found that improvements in depressive symptoms were sustained for up to 12 months. TH programmes may be useful in the prevention of depression. For example, although Perrin et al. [47] did not find a statistically significant reduction in depression, they suggest that the telehealth intervention may have helped to prevent an increase in depressive symptoms in those ICs who did not suffer from depression at baseline. Pierce et al. [46] found that ICs did not increase depressive symptoms after 1 year of web-based intervention; however, they suggested that the main reason was that the ICs were not depressed at baseline.

There were also inconsistent results on caregiver mastery and preparedness, with statistically significant differences found in some studies [44,46,53,57,59] but not in others [43,48,50].

The effects of the TH in the lives of ICs have also been studied, and it has been found that TH reduces the number of unhealthy days [52] and anxiety [54] and improves the psychological domain of the SF-36 [61], the functional independence, and the family functioning [51]. The TH helps also the CIs to modify their lifestyle [60]. The TH has also been found to have a positive effect on task difficulty perception [45] and threat appraisal [45]. However, TH has not shown positive effects on IC satisfaction [43,44,46,47].

An important point to consider in TH programmes is the real needs of the ICs. It has been shown that the needs identified by ICs differ from those perceived by health professionals and/or those designing HT tools [65,74]. If TH programmes are not aligned with the real needs of end-users, their use and adherence will be hampered [75]. For this reason, it is necessary to involve all of them in the design of health technology tools [65].

No adverse effects have been found in any case, showing that TH programmes may be at least as appropriate as the usual care received by ICs of SS.

### 4.5. Risk of Bias

Almost half of the reviewed articles have a low risk of bias [48,49,50,51,52,53,55,57,59]. Of the studies with some concerns, most of them are related to the domain “measurement of the outcome” [44,45,46,47,54,56,58,60,61]. The main limitation is the unblinding of the outcome assessors. LeLaurin et al. [55] evaluated the blinding procedure in their pilot clinical trial. The assessors were asked which group they thought the clinical trial participants had been allocated to. There was a significant association between the pattern of responses for group allocation and the guess of the data collectors. This could be due to participants commenting in a way that could alert the assessors to the intervention that the caregivers were receiving. It is therefore necessary to monitor the correct blinding of the evaluators.

### 4.6. Quality Assessment

Only three of the articles [43,44,54] included in the systematic review had a score of less than 3 on the JADAD scale. In those studies with good methodological quality, the main problem was the lack of double blinding [45,46,47,48,49,50,51,52,53,55,56,57,58,59,60,61]. Given the characteristics of the interventions, it is difficult to achieve blinding of both researchers and participants.

### 4.7. Limitations

This review contributes to improving the knowledge about the TH in ICs of stroke patients. However, there is a potential for selection bias because we did not include conference abstracts or any other grey literature. We have tried to include as many studies as possible; we have screened the references of the papers included in the review and we have searched whether the protocols of the clinical trials found in the databases have been carried out.

Another limitation is that we did not conduct a meta-analysis of the trials included in the review.

There is great variability in the objectives of the articles reviewed, in the intervention programs, and in the outcome measures, which limits the comparability of their results.

Another limitation to be considered is the lack of studies evaluating which of the ST systems is the most appropriate. There were no studies comparing different TH tools. The opinion of the ICs on the most appropriate TH system could be assessed before the development of the TH interventions [76].

There is also no clear evidence on the most appropriate dosage of ST programs. Only LeLaurin et al. [55] compared the feasibility and use of a ST programme with different dosages. In addition, studies of TH that extend interventions over time are rare; only one study [50] was found to maintain the programme for one year. Although the needs of ICs are known at the time of discharge [77], stroke is a chronic condition, and needs change over time [78]. Similarly, no long-term follow-up of outcomes has been found.

The results of this systematic review show that telemedicine can help the ICs of patients who have had a stroke. Although contradictory results were found for some of the variables studied, in no case were the groups receiving telemedicine services shown to have worse outcomes than the control groups, with results at least similar to those of the control interventions [43,44,45,46,47,48,49,50,51,52,53,54,55,56,57,58,59,60,61]. As stroke is a pathology that requires survivor care, which often falls to ICs, it is necessary to consider this type of intervention. In addition, there may be interventions that reduce the cost of caring for these patients, as has been shown in other pathologies [30,79]. However, more controlled clinical trials are needed to investigate the most appropriate tools and doses. It is also necessary to use technologies that are user-friendly and do not require sophisticated equipment. This will help to overcome barriers to technology use [80]. Involving users of these technologies in the development of telehealth programmes can also be an important element in facilitating adherence and success. [81].

## 5. Conclusions

Based on the results of this systematic review, TH may be a useful tool to improve the abilities and health of ICs of SS. Although there are contradictory results on the effects of TH on depression and caregiver burden, positive effects have been found on the health of ICs as well as on their ability to care. No adverse effects have been reported in any of the articles.

More quality studies evaluating the effects of telemedicine on the ICs of stroke survivors are needed. These studies should also assess which tools are most useful, as well as the most appropriate doses.

## Figures and Tables

**Table 1:** Population.

Article	n	Age	Women
Grant et al., 1999 [43]	30 IG: 10 CG: 10 PAG: 10	56 years	21
Grant et al., 2002 [44]	74	56 ± 12 years	67
Bakas et al., 2009 [45]	40 IG: 21 CG: 19	IG: 56.43 ± 9.61 CG: 57.84 ± 11.8	IG: 13 CG: 16
Pierce et al., 2009 [46]	103 IG: 51 CG: 52	IG: 54 ± 12.2 CG: 55 ± 13.1	IG: 25 CG: 30
Perrin et al., 2010 [47]	61	58.5 ± 12.0	56
Smith et al., 2012 [48]	32 IG: 19 CG: 19	IG: 55.3 ± 6.9 CG: 54.9 ± 12.9	NI
Kim et al., 2013 [49]	36 IG: 18 CG: 18	IG: 49.8 ± 14.8 CG: 57.3 ± 11.5	NI
Pfeiffer et al., 2014 [50]	122 IG: 60 CG: 62	IG: 66.7 ± 9.9 CG: 65.6 ± 10.1	IG: 46 CG: 49
Bishop et al., 2014 [51]	49 IG: 23 CG: 26	56.8 ± 16.	32
Bakas et al., 2015 [52]	254 IG: 123 CG: 131	IG: 54.0 ± 12.5 CG: 4.7 ± 11.4	IG: 96 CG: 103
Cheng et al., 2018 [53]	128 IG: 64 CG: 64	IG: 49.08 ± 12.09 CG: 49.11 ± 12.90	IG: 50 CG: 46
Goudarzian et al., 2019 [54]	152 IG: 76 CG: 76	IG: 49.04 ± 14.96 CG: 49.48 (15.05)	90
LeLaurin et al., 2021 [55]	53 IG 4 weeks: 13 IG 8 weeks: 13 CG: 13 AG: 14	60.3 ± 10.1	49
Elsheikh et al., 2022 [56]	110 IG: 55 CG: 55	IG: 35 (25–55) CG: 35 (25–57)	IG: 42 CG: 40
Mou et al., 2022 [57]	40 IG: 20 CG: 20	IG: 45.61 ± 12.14 CG: 48.10 ± 12.20	IG: 9 CG: 10
Demir et al., 2023 [58]	63 IG: 33 CG: 30	IG: 35.48 ± 10.77 CG: 36.07 ± 10.88	IG: 27 CG: 27
Bitek et al., 2023 [59]	80 IG: 40 CG: 40	IG: 54.61 ± 11.73 CG: 51.60 ± 14.18	IG: 70.6% CG: 71.4%
Hussin et al., 2023 [60]	75 IG: 38 CG: 37	IG: 46.1 ± 11.3 CG: 45.6 ± 12.8	IG: 25 CG: 24
Mohammadi et al., 2023 [61]	84 IG: 42 CG: 42	IG: 46.16 ± 11.32 CG: 46.1 ± 10.5	IG: 27 CG: 25

Population characteristics. AG: attention group; CG: control group; IG: intervention group; NI: no information; PAG: personal attention group.

**Table 2:** Interventions (both in the intervention and control groups), main outcome measures, and follow-up carried out in the articles reviewed.

Article	Interventions	Outcomes	Measurement Times
Grant et al., 1999 [43]	IG: telephone contact CG: control PAG: home visit 12 weeks	General health (SF-36) Problem-solving skills (PSI) Satisfaction (CSQ) Depression (CES-D) Caregiver preparedness (PCS) Caregiving burden (CBS)	Week 0 Week 2 Week 5 Week 13
Grant et al., 2002 [44]	IG: social problem-solving telephone partnerships CG: usual discharge planning services SG: sham telephone intervention 12 weeks	General health (SF-36) Problem-solving skills (PSI) Satisfaction (CSQ) Depression (CES-D) Caregiver preparedness (PCS) Caregiving burden (CBS)	Week 0 Week 2 Week 5 Week 13
Bakas et al., 2009 [45]	IG: TASK (phone intervention) CG: attention control group (including phone calls) 8 weeks	Caregiver optimism (LOT-R) Perceived difficulty with tasks (OCBS) Threat appraisal (ACS) Depression (PHQ-9) Caregiver life changes (BCOS) General health (SF-36)	Week 0 Week 4 Week 8 Week 12
Pierce et al., 2009 [46]	IG: web-based intervention CG: usual care 12 months	Depression (CES-D) Satisfaction (SWLS)	Week 0 Month 3 Month 6 Month 9 Month 12
Perrin et a. 2010 [47]	IG: transition assistance program, including 4 videophones CG: standard care 6 weeks	Caregiver burden (CSI) Depression (CES-D) Caregiver satisfaction (VACCHTPSS)	Week 0 Week 4 Week 12
Smith et al., 2012 [48]	IG: web-based intervention (online information, educational videos, chat sessions, e-mail) CG: relevant online information 11 weeks	Depression (CES-D and PHQ-9) Mastery (Mastery Scale) Self esteem (SES) Social support (MOS social support survey) Treatment credibility, reported effort, and perceived benefit	Week 0 Week 11 Week 15
Kim et al., 2013 [49]	IG: web-based intervention CG: usual care 9 weeks	Caregiver mastery (CGMS)	Week 0 Month 3
Pfeiffer et al., 2014 [50]	IG: telephone-based problem-solving intervention CG: information letters 3 months	Depression (CES-D) Caregiving competence (SCQ)	Week 0 Month 3 Month 12
Bishop et al., 2014 [51]	FIIT: family intervention telephone tracking CG: standard medical follow-up 6 months	Functional independence (FAI) Depression (GDS) Family functioning (FAD)	Week 0 Month 3 Month 6
Bakas et al., 2015 [52]	IG: TASK II (phone intervention) CG: information, support, and referral (phone) 12 weeks	Depression (PHQ-9) Caregiver life changes (BCOS) Unhealthy days	Week 0 Week 8 Week 12 Week 24 Week 52
Cheng et al., 2018 [53]	IG: strength-oriented psychoeducational programme (six 30 min phone sessions) CG: usual care 26 weeks	Caregiving competence (CGS) Caregiver burden (CSI) Problem-solving skills (PSI) Depression (CES-D) General health (SF-12) Social support (SSQ)	Week 0 Week 26 Week 32 Week 40
Goudarzian et al., 2019 [54]	IG: phone consultation CG: usual care 3 moths	Depression (BDI) Anxiety (BAI)	Week 0 Month 3
LeLaurin et al., 2021 [55]	IG: RESCUE programme: web-based and phone intervention CG: usual care 4–8 weeks	Depression (CES-D) Caregiver burden (ZBISF)	Week 0 Week 5 or 9 Week 21 or 25
Elsheikh et al., 2022 [56]	IG: multiple methods, including 6 phone calls CG: institutional booklet 6 months	Quality of life (WHOQOL-BREF) Care burden (ZBISF)	Week 0 Month 3 Month 6
Mou et al., 2002 [57]	IG: patient–caregiver dyads education (telephone calls) CG: usual care 4 weeks	Caregiver burden (CSI) Family functioning (F-COPES) Caregiving competence (CCS) Anxiety (GAD-7)	Week 0 Week 4
Betik et al., 2023 [58]	IG: discharge training and telephone counselling (4 sessions) CG: routine care 12 weeks	Caregiver burden (ZCBS)	Week 0 Month 3
Demir et al., 2023 [59]	IG: transitional care model (education via web and phone calls) CG: usual care 12 weeks CG: routine care 12 weeks	Caregiving competence (CCS) Caregiver preparedness (PCS) Caregivers’ e-Health Literacy (eHLS) Caregiver burden (MBIGF)	Week 0 Week 12
Hussin et al., 2023 [60]	IG: stroke riskometer application CG: usual care 12 weeks	Lifestyle changes (LS7) Caregiver’s stroke risk	Week 0 Week 12
Mohammadi et al., 2023 [61]	IG: phone and social media CG: usual care 12 weeks	General health (SF-36)	Week 0 Week 12

ACS: Appraisal of Caregiving Threat Subscale; BAI: Beck Anxiety Inventory; BCOS: Bakas Caregiving Outcomes Scale; BDI: Beck Depression Index; CBS: Caregiving Burden Scale; CG: control group; CCS: Caregiving Competence Scale; CES-D: Centre For Epidemiologic Studies Depression Scale; CGMS: Caregiving Mastery Scale; CSI: Caregiver Strain Index; CSQ: Client Satisfaction Questionnaire; eHLS: e-Health Literacy Scale; F-COPES: Family Crisis-oriented Personal Evaluation Scale; FAD: Family assessment device; FAI: Frenchay activities index; GAD-7: Generalised anxiety disorder scale-7; GDS: geriatric depression scale; IG: intervention group; LS7: Lifestyle 7 scores; LOT-R: revised life orientation test; MBIGF: Maslach Burnout Inventory-General Form; OCBS: Oberst Caregiving Burden Scale Difficulty Subscale; PAG: personal attention group; PCS: Preparedness For Caregiving Scale; PHQ-9: Patient Health Questionnaire Depression Scale; PSI: problem-solving inventory; SES: Self-esteem Scale; SSQ: Six-item Social Support Questionnaire; SWLS: Satisfaction with Life Scale; TASK: Telephone Assessment and Skill Building Kit; VACCHTPSS: VA Care Coordination and Home Telehealth Patient Satisfaction Survey; WHOQOL-BREF: WHO Quality of Life-BREF; ZBISF: Zarit Burden Interview-Short Form; ZCBS: Zarit Caregiver Burden Scale.

**Table 3:** Main results of the articles reviewed.

Article	Outcomes	Results
Grant et al., 1999 [43]	CES-D week 2 = ß = 1.63 (*p* < 0.01) CES-D week 5 = ß = 0.91 (*p* = 0.05) PSI week 2 = ß = −2.26 (*p* < 0.01) PSI week 5 = ß = −2.14 (*p* < 0.05) PCS week 1 = ß = 1.36 (*p* < 0.01) PCS week 5 = ß = 0.73 (*p* < 0.05)	The IG scored statistically significantly better on depression, problem-solving skills, and caregiving preparedness during the intervention (weeks 2 and 5) compared to the other two groups. It also improved, although without significant results, after the intervention (weeks 13).
Grant et al., 2002 [44]	PCS = IG = 19.12 ± 1.61 (*p* < 0.01); CG = 0.01 ± 1.65 (*p* > 0.05); SG = 0.78 ± 1.63 (*p* > 0.05) CES-D = IG = 14.68 ± 1.61 (*p* < 0.01); CG = 0.08 ± 1.65 (*p* > 0.05); SG = 3.56 ± 1.63 (*p* > 0.05) PSI = IG = −7.2 ± 1.59 (*p* < 0.05); GC = 1.91 ± 1.65 (*p* > 0.05); SG = 0. ± 1.62 (*p* > 0.05)	The IG showed better problem-solving skills, greater caregiver preparedness, and less depression compared to SG and CG (*p* < 0.05). No differences were found in the caregiving burden.
Bakas et al., 2009 [45]	LOT-R week 4 = 18.34 ± 0.92 vs. 14.78 ± 0.97 (*p* < 0.05) LOT-R week 8 = 18.14 ± 0.84 vs. 14.85 ± 0.89 (*p* < 0.05) LOT-R week 12 = 17.61 ± 1 vs. 13.59 ± 1.06 (*p* < 0.05) OCBS week 4 = 22.87 ± 1.38 vs. 27.94 ± 1.47 (*p* < 0.05) OCBS week 8 = 22.61 ± 1.39 vs. 26.07 ± 1.48 (*p* > 0.05) OCBS week 12 = 22.34 ± 1.41 vs. 24.63 ± 1.49 (*p* > 0.05) ACS week 4 = 31.55 ± 1.83 vs. 34.5 ± 1.94 (*p* > 0.05) ACS week 8 = 28.38 ± 1.86 vs. 35.48 ± 1.98 (*p* < 0.05) ACS week 12 = 30.64 ± 2.84 vs. 38.92 ± 1.87 (*p* < 0.05)	The IG showed significant increases in optimism (weeks 4, 8, 12), improvement in task difficulty (week 4), and threat appraisal (weeks 8 and 12) compared to the CG.
Pierce et al., 2009 [46]	CES-D = 12.3 ± 9.8 vs. 9 ± 9.1 (*p* > 0.05) SWLS = 21.7± 6.7 vs. 24.6± 6 (*p* > 0.05)	No statistical difference between groups was found in depression and satisfaction.
Perrin et al., 2010 [47]	CSI 1–3 months ^a^ = −1.64 ± 3.31 vs. 2 ± 6.26 (*p* < 0.05) CES-D = −1.81 ^b^ (*p* > 0.05)	The IG shows significantly lower caregiver strain at 3 months. The IG showed also less depression than the CG, but without statistical significance.
Smith et al., 2012 [48]	CES-D week 11 = 13.9 ± 2 vs. 19.7 ± 1.8 (*p* < 0.05) CES-D week 15 = 13.4 ± 1.6 vs. 24.1 ± 0.5 (*p* < 0.05)	The IG showed significantly lower depression than the CG in weeks 11 and 15. No significant differences were found for other variables.
Kim et al., 2013 [49]	CGMS = (19.7 ± 2.8 vs. 22.8 ± 2.5) (*p* < 0.05)	The IG improved significantly in mastery.
Pfeiffer et al., 2014 [50]	CES-D months 3 = 17.3 ± 7.55 vs. 20.4 ± 9.44 (*p* < 0.05) CES-D months 12 = 2.4 ± 7.52 vs. 18.2 ± 10.87 (*p* < 0.05)	The IG showed significantly lower levels of depressive symptoms after both 3 and 12 months. No significant differences were found for other variables.
Bishop et al., 2014 [51]	FAD months 3 = 2.4 ± 4.6 vs. −2.5 ± 3.5 (*p* < 0.05) FAD month 6 = 2.7 ± 6.4 vs. −2.8 ± 4 (*p* < 0.05) FAI month 3 = −0.65 ± 5.4 vs. 2.13 ± 3.72 (*p* < 0.05) FAI month 6 = −0.84 ± 4.5 vs. 1.74 ± 3.8 (*p* < 0.05)	The IG showed an improvement in both independence functioning in months 3 and 6 and family functioning in months 3 and 6.
Bakas et al., 2015 [52]	PHQ-9 weeks 1–8 = −3.6 ± 0.8 vs. −0.9 ± 0.7 (*p* < 0.05) PHQ.9 weeks 1–12= −3.9 ± 0.8 vs. −2 ± 0.7 (*p* < 0.05) PHQ-9 weeks 1–24 = −3.6 ± 0.7 vs. −1.6 ± 0.6 (*p* < 0.05) PHQ-9 weeks 1–52 = −4 ± 0.8 vs. −1.1 ± 0.7 (*p* < 0.05) BCOS weeks 1–8 = 2.9 ± 1.3 vs. 1.2 ± 1.2 (*p* < 0.05) Unhealthy days weeks 1–8 = −1.1 ± 0.9 vs. 1.8 ± 0.9 (*p* < 0.05)	There was a statistically significant reduction in depression in weeks 8, 12, 24, and 52; an improvement in life changes in week 8 and a reduction in unhealthy days in week 8 in the IG.
Cheng et al., 2018 [53]	CGS week 26 = 12.02 ± 1.79 vs. 11.16 ± 2.35 CGS week 32 = 12.31 ± 1.46 vs. 10.65 ± 2.07 CGS week 40 = 12.48 ± 1.28 vs. 10.65 ± 2.10 (*p* < 0.05) PSI week 26 = 94.69 ± 12.56 vs. 103.67 ± 19.08 (*p* < 0.05) PSI week 32 = 93.96 ± 13.89 vs. 106.70 ± 20.12 (*p* < 0.05) PSI week 40 = 92.59 ± 10.82 vs. 108.08 ± 18.61 (*p* < 0.05) SSQ week 26 = 5.15 ± 0.55 vs. 4.90 ± 0.84 (*p* < 0.05) SSQ week 32 = 5.23 ± 0.51 vs. 4.83 ± 0.88 (*p* < 0.05) SSQ week 40 = 5.11 ± 0.49 vs. 4.81 ± 0.80 (*p* < 0.05)	The IG improved, compared to CG, throughout the study in caregiving competence problem-solving coping abilities (T0–T1: −5.93 (−11.08, −6.81); T0–T2: −8.74 (−13.81, −3.67); T0–T3: −12.34 (−17.88, −6.81) and social support satisfaction (T0–T1: 0.28 (0.08, 0.47); T0–T1: 0.42 (0.20, 0.64); T0–T3: 0.33 (0.10, 0.55) (*p* < 0.01).
Goudarzian et al., 2019 [54]	BAI = 30.18 ± 5.53 vs. 33.59 ± 6.4 (*p* < 0.05) BDI = 35.41 ± 9.34 vs. 35.85 ± 7.8 (*p* > 0.05)	The IG statistically improved in anxiety, but not (*p* > 0.05) in depression.
LeLaurin et al., 2021 [55]	CES-D week 5/9 = 9.4 ± 6.1 vs. 16.4 ± 8.6 vs. 16.2 ± 10.4 vs. 13.7 ± 12.5 CES-D week 21/25 = 12 ± 6.3 vs. 12.9 ± 10.4 vs. 12.7 ± 11.1 vs. 11.6 ± 10.7 ZBISF week 5/9 = 9.4 ± 6.1 vs. 16.4 ± 8.6 vs. 16.2 ± 10.4 vs. 13.7 ± 12.5 ZBISF week 21/25 = 12.6 ± 12.4 vs. 12.9 ± 10.4 vs. 12.7 ± 11.1 vs. 11.6 ± 10.7	Although the study was not powered for significance testing, no statistically significant findings were found.
Elsheikh et al., 2022 [56]	WHOQOL-BREF psychological month 3 = 43.41 ± 19.93 vs. 42.73 ± 18.89 WHOQOL-BREF psychological month 6 = 43.86 ± 19.88 vs. 42.35 ± 19.04 WHOQOL-BREF social month 3 = 71.06 ± 15.45 vs. 70.91 ± 14.77 WHOQOL-BREF social month 6 = 72.12 ± 15.57 vs. 69.85 ± 16.08 ZBISF month 3 = 34.38 ± 7.09 vs. 32.53 ± 7.96 (*p* > 0.05) ZBISF month 6 = 34.60 ± 7.07 vs. 33.24 ± 7.83 (*p* > 0.05)	Although the effects of group and time interaction on both the psychological and social relationship domains were significant (*p* < 0.05), no significant differences within groups or between groups for all domains of QoL were found. No differences were found between groups in care burden.
Mou et al., 2022 [57]	CBI = 32.45 ± 15.86 vs. 44.4 ± 16.5 (*p* < 0.05) CCS = 11.75 ± 2.51 vs. 11.4 ± 1.35 (*p* < 0.05) F-COPES = 98.75 ± 7.65 vs. 93.4 ± 8.52 (*p* > 0.05) GAD-7 = 3.45 ± 3.39 vs. 4.65 ± 4.07 (*p* > 0.05)	The caregiver burden was significantly reduced in the IG compared to the CG. The IG also improved caregivers’ competence.
Bitek et al., 2023 [58]	ZCBS = 32.14 ± 15.4 vs. 34.27 ± 14.2 (*p* < 0.05)	The caregiver´s burden was significantly lower in the IG compared to the CG after the intervention.
Demir et al., 2023 [59]	CCS = 13.48 ± 2.31 vs. 11.37 ± 2.48 (*p* < 0.001) PCS = 28.48 ± 4.74 vs. 20.93 ± 7.10 (*p* < 0.001) eHLS = 34.42 ± 4.74 vs. 26.93 ± 8.53 (*p* < 0.001) MBIGF emotional exhaustion = 7.24 ± 3.27 vs. 8.90 ± 3.58 (*p* < 0.05) MBIGF personal accomplishment = 10.45 ± 4.13 vs. 16.93 ± 5.10 (*p* < 0.05) MBIGF depersonalization= 6.03 ± 2.38 vs. 6.43 ± 2.70 (*p* > 0.05)	After the intervention, the IG exhibited significantly better caregiver competence, preparation for care, and e-health literacy than the CG. The IG showed also better results in emotional exhaustion and personal accomplishment.
Hussin et al., 2023 [60]	LS7 ^c^ = 9.29 (1.59) vs. 8.41 (1.87) (*p* < 0.05) Stroke risk 5 years ^c^ = 2.04 (1.21) vs. 2.57 (1.70) (*p* > 0.05) Stroke risk 10 years ^c^ = 3.53 (2.50) vs. 4.34 (3.28) (*p* > 0.05)	The IG showed a better improvement in LS7 than CG at 3 months (median difference = (95% CI) = 0.88 (1.68–0.08) (*p* < 0.05). No differences were found in the risk of stroke (*p* > 0.05).
Mohammadi et al., 2023 [61]	SF-36 psychological subscale = 64.4 ± 14.53 vs. 51.09 ± 14.07 (*p* < 0.05) SF-36 physical subscale = 80.98 ± 17.06 vs. 77.71 ± 15.21 (*p* > 0.05)	The IG showed significant differences in the psychological subscale of the SF-36 compared to the CG, but not in the physical domain.

ACS: Appraisal of Caregiving Threat Subscale; BAI: Beck Anxiety Inventory; BCOS: Bakas Caregiving Outcomes Scale; BDI: Beck Depression Index; CBI: Caregiver Burden Inventory; CBS: Caregiving Burden Scale; CCS: Caregiving Competence Scale; CES-D: Centre For Epidemiologic Studies Depression Scale; CGMS: Caregiving Mastery Scale; CGS: Caregiving competence; CSI: Caregiver Strain Index; ; eHLS: e-Health Literacy Scale; F-COPES: Family Crisis-oriented Personal Evaluation Scale; FAD: Family assessment device; FAI: Frenchay activities index; GAD-7: Generalised anxiety disorder scale-7; LOT-R: revised life orientation test; LS7: Lifestyle 7 scores; MBIGF: Maslach Burnout Inventory-General Form; OCBS: Oberst Caregiving Burden Scale Difficulty Subscale; PCS: Preparedness For Caregiving Scale; PHQ-9: Patient Health Questionnaire Depression Scale; PSI: problem-solving inventory; SSQ: Six-item Social Support Questionnaire; SWLS: Satisfaction with Life Scale; WHOQOL-BREF: WHO Quality of Life-BREF; ZBISF: Zarit Burden Interview-Short Form; ZCBS: Zarit Caregiver Burden Scale. ^a^: mean differences (standard deviation); ^b^: *t*-statistic; ^c^: medians (interquartile range).

## Data Availability

Not applicable.

## Appendix A

Search strategy: Pubmed

((“stroke”[MeSH Terms] OR “stroke”[All Fields] OR “strokes”[All Fields] OR “stroke s”[All Fields] OR (“cerebrovascular”[All Fields] AND “accident”[All Fields]) OR “cerebrovascular accident”[All Fields] OR “cva”[All Fields] OR (“cerebrovascular”[All Fields] AND (“event”[All Fields] OR “event s”[All Fields] OR “events”[All Fields])) OR “cve”[All Fields])

AND

(“caregiver s”[All Fields] OR “caregivers”[MeSH Terms] OR “caregivers”[All Fields] OR “caregiver”[All Fields] OR “caregiving”[All Fields] OR (“informal”[All Fields] AND “caregiver”[All Fields]) OR “informal caregiver”[All Fields] OR “family”[MeSH Terms] OR “family”[All Fields] OR “relative”[All Fields] OR “relatives”[All Fields] OR “relative s”[All Fields] OR “relatively”[All Fields] OR (“family”[All Fields] AND “members”[All Fields]) OR “family members”[All Fields] OR “caregiver burden” [MeSH Terms] OR (“caregiver”[All Fields] AND “burden”[All Fields]) OR “caregiver burden”[All Fields] OR (“caregiver”[All Fields] AND “stress”[All Fields]) OR “caregiver stress”[All Fields] OR “caregiver fatigue”[All Fields] OR ((“caregiver s”[All Fields] OR “caregivers”[MeSH Terms] OR “caregivers”[All Fields] OR “caregiver”[All Fields] OR “caregiving”[All Fields]) AND (“fatiguability”[All Fields] OR “fatiguable”[All Fields] OR “fatigue”[MeSH Terms] OR “fatigue”[All Fields] OR “fatigued”[All Fields] OR “fatigues”[All Fields] OR “fatiguing”[All Fields] OR “fatigability”[All Fields])) OR (“caregiver”[All Fields] AND “burden”[All Fields]) OR “caregiver burden”[All Fields] OR (“caregiver”[All Fields] AND “burnout”[All Fields]) OR “caregiver burnout”[All Fields]))

AND

(“mobile applications”[MeSH Terms] OR (“mobile”[All Fields] AND “applications”[All Fields]) OR “mobile applications”[All Fields] OR (“mobile”[All Fields] AND “application”[All Fields]) OR “mobile application”[All Fields] OR (“mobile”[All Fields] AND “app”[All Fields]) OR “mobile app”[All Fields] OR “mhealth s”[All Fields] OR “telemedicine”[MeSH Terms] OR “telemedicine”[All Fields] OR “mhealth”[All Fields] OR “ehealth”[All Fields] OR “internet based intervention”[MeSH Terms] OR (“internet based”[All Fields] AND “intervention”[All Fields]) OR “internet based intervention”[All Fields] OR (“web”[All Fields] AND “based”[All Fields] AND “intervention”[All Fields]) OR “web based intervention”[All Fields] OR (“internet”[All Fields] AND “based”[All Fields] AND “intervention”[All Fields]) OR “internet based intervention”[All Fields] OR “telehealth s”[All Fields] OR “telehealth”[All Fields] OR “telemonitor”[All Fields] OR “telemonitored”[All Fields] OR “telemonitoring”[All Fields] OR “telemonitors”[All Fields] OR “telepractice”[All Fields] OR “telenursing”[MeSH Terms] OR “telenursing”[All Fields] OR “telecare”[All Fields] OR “computers, handheld”[MeSH Terms] OR (“computers”[All Fields] AND “handheld”[All Fields]) OR “handheld computers”[All Fields] OR (“computer”[All Fields] AND “handheld”[All Fields]) OR “computer handheld”[All Fields] OR “telerehabilitation”[MeSH Terms] OR “telerehabilitation”[All Fields] OR (“tele”[All Fields] AND “rehabilitation”[All Fields]) OR “tele rehabilitation”[All Fields] OR (“virtual”[All Fields] AND “rehabilitation”[All Fields]) OR “virtual rehabilitation”[All Fields] OR (“remote”[All Fields] AND “rehabilitation”[All Fields]) OR “remote rehabilitation”[All Fields] OR “mobile applications”[MeSH Terms] OR (“mobile”[All Fields] AND “applications”[All Fields]) OR “mobile applications”[All Fields] OR (“mobile”[All Fields] AND “app”[All Fields]) OR “mobile app”[All Fields] OR (“mobile”[All Fields] AND “application”[All Fields]) OR “mobile application”[All Fields] OR “smartphone”[MeSH Terms] OR “smartphone”[All Fields] OR “smartphones”[All Fields] OR “smartphone s”[All Fields] OR “cell phone”[MeSH Terms] OR (“cell”[All Fields] AND “phone”[All Fields]) OR “cell phone”[All Fields] OR “telephone”[MeSH Terms] OR “telephone”[All Fields] OR “telephones”[All Fields] OR “telephoned”[All Fields] OR “telephonic”[All Fields] OR “telephonically”[All Fields] OR “telephoning”[All Fields] OR “hotlines”[MeSH Terms] OR “hotlines”[All Fields] OR (“hot”[All Fields] AND “lines”[All Fields]) OR “hot lines”[All Fields] OR “patient portals”[MeSH Terms] OR (“patient”[All Fields] AND “portals”[All Fields]) OR “patient portals”[All Fields] OR “videoconferenced”[All Fields] OR “videoconferencing”[MeSH Terms] OR “videoconferencing”[All Fields])

Search strategy: Scopus

TITLE-ABS-KEY ((“stroke*” OR “cerebrovascular accident” OR “cva” OR “cerebrovascular event” OR “cve”) AND (“caregiv*” OR “informal caregiv*” OR “famil* caregiv*” OR “relativ* caregiv*” OR “caregiv* burden” OR “caregiv* stress” OR “caregiv* fatigu*” OR “caregiv* burnout”) AND (“mobile app*” OR “mhealth*” OR “telemedicine” OR “ehealth*” OR “internet based intervention*” OR “web based intervention*” OR “telehealth*” OR “telemonitor*” OR “telepractic*” OR “telenurs*” OR “telecare” OR “handheld computer*” OR “telerehabilitation” OR “virtual rehabilitation” OR “remote rehabilitation” OR “Smartphone*” OR “cell phone*” OR “telephon*” OR “hot line*” OR “patient* portal*” OR “videoconferenc*”))

Search strategy: Web of Science

(TS = stroke* OR TS = cerebrovascular accident OR TS = cva OR TS = cerebrovascular event* OR TS = cve) AND (TS = caregiv* burden OR TS = caregiv* stress OR TS = caregiv* fatigu* OR TS= caregiv* burnout) AND (TS = mobile app* OR TS = mhealth* OR TS = telemedicine OR TS = ehealth* OR TS = internet based intervention* OR TS = web based intervention* OR TS = telehealth* OR TS = telemonitor* OR TS = telepractic* OR TS = telenurs* OR TS = telecare OR TS = handheld computer* OR TS = telerehabilitation OR TS = virtual rehabilitation OR TS = remote rehabilitation OR TS = Smartphone* OR TS = cell phone* OR TS = telephon* OR TS = hot line* OR TS = patient* portal* OR TS = videoconferenc*)

Search strategy: CINAHL, Psychology and Behavioral Sciences Collection, APA PsycInfo

(“stroke*” OR “cerebrovascular accident” OR “cva” OR “cerebrovascular event” OR “cve”) AND (“caregiv*” OR “informal caregiv*” OR “famil* caregiv*” OR “relativ* caregiv*” OR “caregiv* burden” OR “caregiv* stress” OR “caregiv* fatigu*” OR “caregiv* burnout”) AND (“mobile app*” OR “mhealth*” OR “telemedicine” OR “ehealth*” OR “internet based intervention*” OR “web based intervention*” OR “telehealth*” OR “telemonitor*” OR “telepractic*” OR “telenurs*” OR “telecare” OR “handheld computer*” OR “telerehabilitation” OR “virtual rehabilitation” OR “remote rehabilitation” OR “Smartphone*” OR “cell phone*” OR “telephon*” OR “hot line*” OR “patient* portal*” OR “videoconferenc*”).

## Appendix B

**Table A1:** JADAD scale for each article.

Paper	Randomisation Present	Appropriated Randomisation Utilised	Blinded Present	Appropriated Blinded Utilised	Description of Withdrawals and Dropouts	Total
Grant et al., 1999 [43]	0	0	0	0	0	0
Grant et al., 2002 [44]	1	0	0	0	1	2
Bakas et al., 2009 [45]	1	1	0	0	1	3
Pierce et al., 2009 [46]	1	1	0	0	1	3
Perrin et al., 2010 [47]	1	1	0	0	1	3
Smith et al., 2012 [48]	1	1	0	0	1	3
Kim et al., 2013 [49]	1	1	0	0	1	3
Pfeiffer et al., 2014 [50]	1	1	0	0	1	3
Bishop et al., 2014 [51]	1	1	0	0	1	3
Bakas et al., 2015 [52]	1	1	0	0	1	3
Cheng et al., 2015 [53]	1	1	0	0	1	3
Goudarzian et al., 2019 [54]	1	1	0	0	0	2
LeLaurin et al., 2021 [55]	1	1	0	0	1	3
Elsheikh et al., 2022 [56]	1	1	0	0	1	3
Mou et al., 2022 [57]	1	1	0	0	1	3
Betik et al., 2023 [58]	1	1	0	0	1	3
Demir et al., 2023 [59]	1	1	0	0	1	3
Hussin et al., 2023 [60]						
Mohammadi et al., 2023 [61]	1	1	0	0	1	3
